# Inhibition of Protein Aggregation: Supramolecular Assemblies of Arginine Hold the Key

**DOI:** 10.1371/journal.pone.0001176

**Published:** 2007-11-14

**Authors:** Utpal Das, Gururao Hariprasad, Abdul S. Ethayathulla, Pallavi Manral, Taposh K. Das, Santosh Pasha, Anita Mann, Munia Ganguli, Amit K. Verma, Rajiv Bhat, Sanjeev Kumar Chandrayan, Shubbir Ahmed, Sujata Sharma, Punit Kaur, Tej P. Singh, Alagiri Srinivasan

**Affiliations:** 1 Department of Biophysics, All India Institute of Medical Sciences, Ansari Nagar, New Delhi, India; 2 Department of Anatomy, All India Institute of Medical Sciences, Ansari Nagar, New Delhi, India; 3 Peptide Chemistry Laboratory, Institute of Genomics and Integrative Biology, University of Delhi, Delhi, India; 4 Atomic Force Microscopy (AFM) Laboratory, Institute of Genomics and Integrative Biology, University of Delhi, Delhi, India; 5 School of Biotechnology, Center for Biotechnology, Jawaharlal Nehru University, New Delhi, India; 6 Division of Protein Science and Engineering, Institute of Microbial Technology, Chandigarh, India; Fred Hutchinson Cancer Research Center, United States of America

## Abstract

**Background:**

Aggregation of unfolded proteins occurs mainly through the exposed hydrophobic surfaces. Any mechanism of inhibition of this aggregation should explain the prevention of these hydrophobic interactions. Though arginine is prevalently used as an aggregation suppressor, its mechanism of action is not clearly understood. We propose a mechanism based on the hydrophobic interactions of arginine.

**Methodology:**

We have analyzed arginine solution for its hydrotropic effect by pyrene solubility and the presence of hydrophobic environment by 1-anilino-8-naphthalene sulfonic acid fluorescence. Mass spectroscopic analyses show that arginine forms molecular clusters in the gas phase and the cluster composition is dependent on the solution conditions. Light scattering studies indicate that arginine exists as clusters in solution. In the presence of arginine, the reverse phase chromatographic elution profile of Alzheimer's amyloid beta 1-42 (Aβ_1-42_) peptide is modified. Changes in the hydrodynamic volume of Aβ_1-42_ in the presence of arginine measured by size exclusion chromatography show that arginine binds to Aβ_1-42_. Arginine increases the solubility of Aβ_1-42_ peptide in aqueous medium. It decreases the aggregation of Aβ_1-42_ as observed by atomic force microscopy.

**Conclusions:**

Based on our experimental results we propose that molecular clusters of arginine in aqueous solutions display a hydrophobic surface by the alignment of its three methylene groups. The hydrophobic surfaces present on the proteins interact with the hydrophobic surface presented by the arginine clusters. The masking of hydrophobic surface inhibits protein-protein aggregation. This mechanism is also responsible for the hydrotropic effect of arginine on various compounds. It is also explained why other amino acids fail to inhibit the protein aggregation.

## Introduction

Understanding protein aggregation during refolding and expression of proteins in heterologous systems is an important area in basic research as well as in pharmaceutical industry. Protein aggregation is also thought to be associated with several disease processes. It is generally observed that proteins tend to aggregate during in vitro refolding of proteins when the denaturant is being removed [1]. The non-polar residues exposed during denaturation mediate this aggregation [2]. The intra-chain interactions lead to specific folding of polypeptide to assume native conformation. The inter-chain interactions lead to protein aggregation. Favoring the kinetic competition toward intra-chain interactions is an important issue for the generation of proteins in native state. At present, there is no general panacea for this problem. Currently, this problem is being dealt with empirically by the addition of solutes and co-solvents to the protein solutions. Solution additives such as amino acids, salts, osmolytes can modify the solution behavior of the proteins [3 and the references therein]; [4]. Many theories have been proposed to explain the effect of these solution additives for the prevention of protein aggregation [5]–[9], [reviewed in 10]. These mechanisms are based on the interaction of additives with proteins (preferential interaction) [7], [10] and amino acids (amino acid solubility) [10] or the effects on water structure (surface tension) [11]. An attempt has been made to design solution additives using ‘gap effect (similar to osmotic stress) [8]. However, this hypothesis cannot differentiate between a denaturant, a solubilizer, a stabilizer and an aggregation suppressor.

Arginine and proline have been consistently shown to be helpful in preventing protein aggregation due to heating, dilution or partial unfolding [12]–[17]. Arginine does not change the equilibrium of the folding process [15], [17], [18]. It only prevents the association of denatured or partially folded protein [19]–[23]. The hydrotropic effect of arginine on fatty acids has also been documented [24]. Experimental results show that arginine shifts the second virial coefficient to the positive side and suppresses aggregation [25], [26]. Though it has been termed as the most polar amino acid, arginine exhibits hydrotropic effect. Its effect has been observed with proteins, peptides and fatty acids. It has been observed that either the surface tension effect or any other parameters discussed earlier cannot explain the effect of arginine [10]. The explanations proposed so far do not clearly distinguish the interactions of arginine with protein and water. It is also not explained how these are different from the interactions of other additives that do not inhibit protein aggregation. All proposed mechanisms do not consider the hydrophobic interactions, which are mainly responsible for the aggregation of unfolded proteins. It has not been experimentally verified whether arginine combines with the protein or peptide involving the exposed hydrophobic region and/or modulates the hydrophobic interactions. It has been suggested that multimeric forms proline may be responsible for its aggregation inhibitory effects [12], [13]. However, there is no direct evidence in these studies to show that the multimeric forms modulate the hydrophobic properties of the protein. To answer these questions, we have chosen the mouse amyloid Aβ_1-42_ peptide as the model system because it is insoluble in aqueous medium and its aggregation pattern due to hydrophobic interactions is characterized. Our results show that arginine is present as molecular clusters in solutions. These clusters present a hydrophobic surface by the alignment of its methylene groups. This hydrophobic surface modulates the hydrophobic behavior and prevents hydrophobic surface induced aggregation by binding to Aβ_1-42_. The results presented here are also the first report of the effects of amino acids on Aβ_1-42_ solubilization and aggregation.

## Results and Discussion

### Arginine solutions present hydrophobic environments

**Figure 1:**
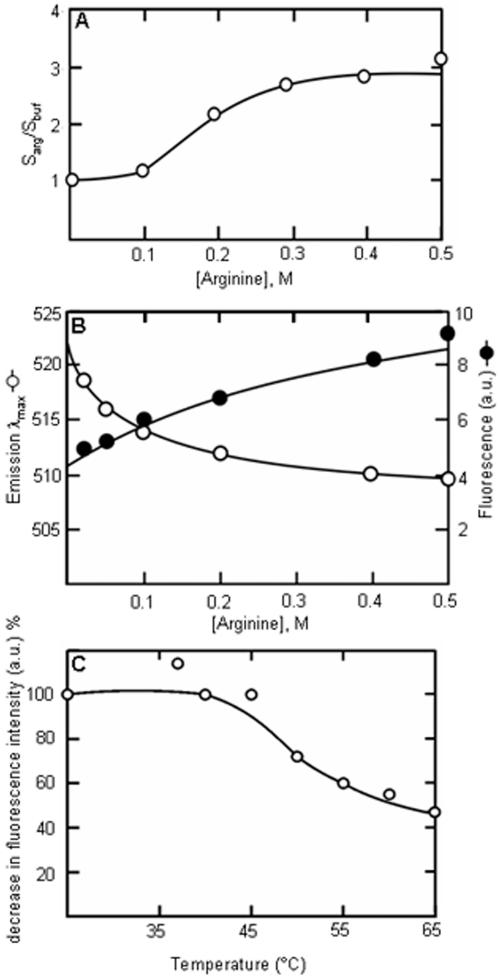
Non-polar environment in arginine solutions. (A) Pyrene solubility in presence of arginine. The solubility is expressed as fold-increase over the control (solubility in arginine/solubility in buffer). 1 mg pyrene was incubated in arginine solutions at the indicated concentrations at 25°C for 24 h. The absorbance of the supernatant solution was measured at 350 nm. The solubility increases in a dose dependent manner. (B) ANS fluorescence in the presence of arginine. The excitation wavelength was 400 nm and the emission intensity was scanned from 450 to 600 nm. With the increase in arginine concentration, the maximum emission wavelength of ANS (250 µM in PB) decreases (open circle) and relative fluorescence intensity increases (closed circle). (C) Temperature dependence of ANS fluorescence in the presence of 0.2 M arginine. The observed intensity is expressed as % of intensity at 25°C. The intensity decreases above 45°C.

The polarity of arginine solutions in 0.02 M sodium phosphate buffer, pH 7.4, (PB) was studied using pyrene solubility and ANS fluorescence characteristics. Pyrene, with its polarity sensing solubility is useful for such studies. Pyrene is sparingly soluble in water. Its solubility increases with the decrease in the polarity of the solvent. Arginine increased the solubility of pyrene in PB in a dose dependent manner (Figure 1A). At 0.5 M arginine concentration, the pyrene solubility increased by three-fold. This hydrotropic effect of arginine has been observed with many other systems as described earlier. However, the mechanism for the hydrotropic effect of arginine is not clear [18], [27]. The part of the arginine molecule, which could be responsible for this effect on the non-polar compounds are its three methylene groups (C^β^, C^γ^ and C^δ^). The hydrophobic interaction of these methylene groups has been observed in other systems as well [28]. This aliphatic side-chain of arginine is shown to interact with the naphthalene [29] or the phenyl [30], [31] ring of ANS. In our experiments, an increase in the intensity of fluorescence emission and a blue shift of the emission λ_max_ has been observed in a concentration dependent manner (Figure 1B). These two changes are characteristics of ANS fluorescence when it is in a hydrophobic environment [32]. Two fold increase in the intensity of fluorescence emission and a blue shift of the emission λ_max_ of 12 nm has been observed with 0.5 M arginine. The hydrotropic effect on pyrene and the ANS fluorescence characteristics indicate that arginine solutions display hydrophobic environment. The interactions resulting in the display of hydrophobic environment are non-covalent in nature and are affected by an increase in solution temperature. Above 45°C, the ANS fluorescence intensity decreased (Figure 1C). The hydrophobic environment of the arginine solutions may interfere with the hydrophobic association of unfolded proteins. Prevented from aggregating, the unfolded proteins remain soluble. The soluble unfolded proteins can fold into native conformation. This would increase the yield of proteins with native conformation.

### Arginine forms molecular clusters

**Figure 2:**
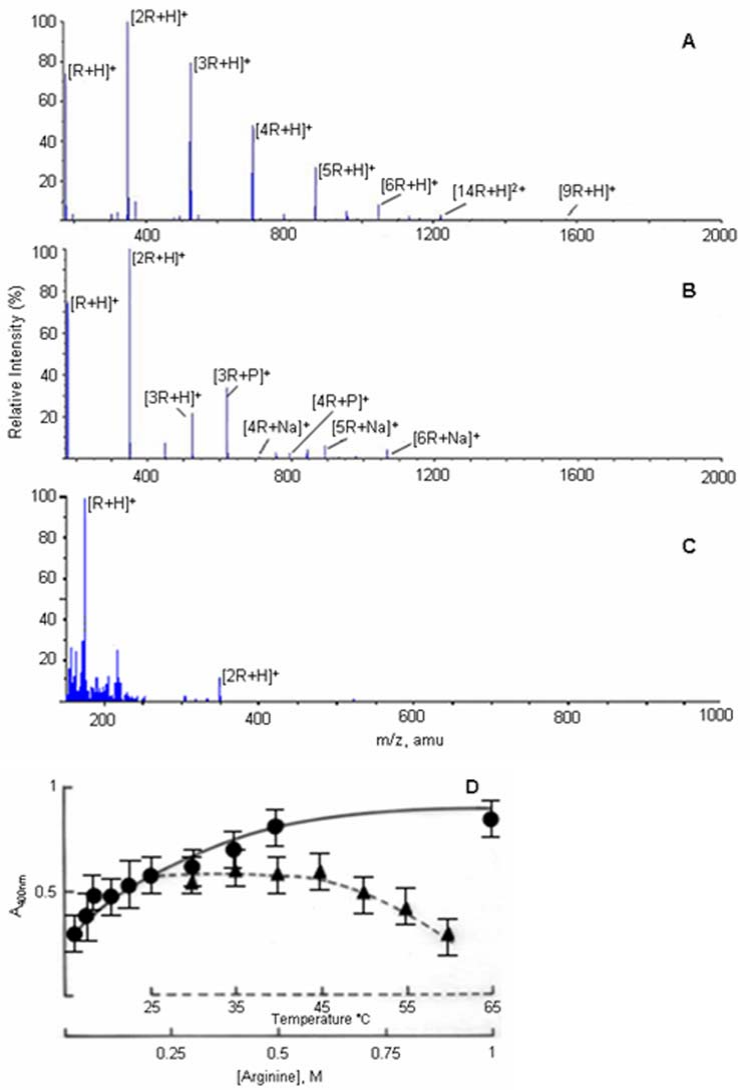
Molecular clusters of arginine in solution. Electrospray mass spectroscopy of amino acids. The aqueous solutions amino acids at 0.2 M concentrations were used. (A) Arginine exhibits extensive noncovalent protonated clusters when dissolved in water, pH without adjustment was 10.5. (B) ([nArg]Na)^+^ and ([nArg]H_2_PO_4_)^+^ clusters are observed when arginine is dissolved in sodium phosphate buffer, pH 7.4. (C) Less extensive clustering is seen in acidic solutions at pH 1.0. (D) Increase in Rayleigh light scattering by arginine solution (in PB) is concentration dependent (filled circle) indicating supramolecular assembly. This assembly is temperature sensitive and collapses above 45°C (filled triangle).

Interaction of arginine with other molecules involves both its polar and non-polar moieties. Experimental evidences using model compounds show that 3–5 arginine molecules are required to bind one ANS molecule and the binding is cooperative [33]. It is probable that ANS binds to an arginine cluster. Mass spectroscopy is a useful technique to analyze the clustering of amino acids. The previous mass spectroscopic studies of amino acids were carried out in predominantly non-polar or acidic conditions [34], [35]. We have studied the clustering of arginine and other amino acids in PB, under conditions used in our experiments. Arginine formed large clusters in the gas phase. The clustering was dependent on the solution conditions (Figure 2). In water solutions, (pH ∼10.5), largely protonated species of arginine were observed (Figure 2A). At pH 7.4, buffered with sodium phosphate, the clusters were associated with sodium and phosphate groups (Figure 2B). At pH 1.0, less clustering was seen indicating that the carboxylate groups were involved in the cluster formation (Figure 2C). The ionic species observed in the gas phase were dependent on the solution conditions. Similar observations have been made using analytical laser induced liquid beam desorption mass spectrometry [35]. Arginine had higher propensity to form clusters than any other amino acids containing aliphatic chain or many methylene groups [[34], Figure S1]. It has been shown that very large clusters of amino acids can be formed extending to nanometer dimensions. It is also evident that chirally pure amino acids tend to cluster in rod-shaped elongated structures [36]. Direct evidence for existence of clusters of arginine in solution was provided by the light scattering experiments. Rayleigh light scattering by arginine in solutions increased in a concentration dependent manner (Figure 2D). However, it appeared to saturate at higher concentrations beyond 0.5 M. Similar results have been observed for proline in the concentration range of 1–2.5 M. Even at low concentrations of arginine, the scattering intensity increased continuously, whereas this effect was seen with proline only at concentrations above 1 M. This observation is in accordance with the observed efficiency of arginine and proline in preventing protein aggregation. The supramolecular assembly due to noncovalent polar interactions, is expected to be temperature sensitive and collapse at higher temperatures. We have seen that the scattering intensity decreased beyond 45°C (Figure 2D), similar to the decrease in the ANS fluorescence intensity. These results show that ANS binds to the hydrophobic surface on the arginine clusters. When the cluster formation is prevented at above 45°C, the ANS fluorescent intensity decreases.

Large molecular clusters in solution resemble crystalline state in the intermolecular interactions, orientation of the molecules, self-salvation, etc. Typically, amino acids orient themselves in a peptide-like fashion with N- and C-terminal groups at juxtapositions and the side chains protruding away on both sides [37]. In contrast, arginine stacks in head-to-tail fashion and a hydrophobic column composed of the three methylene groups is seen along one crystallographic axis [[38], Figure S2]. This orientation and packing is observed in many crystal structures of arginine [39], [40]. This unique property of arginine stems from the strong interactions between their guanidium and carboxylate groups of adjacent molecules. These clusters may have conformational properties as observed in crystal structures and be rod shaped as shown by calculations for chirally pure proline [36]. In arginine clusters, the alignment of C^β^, C^γ^ and C^δ^ would present a hydrophobic surface similar to that seen in the crystals (Figure S2).

### Arginine modulates the hydrophobic interactions of Alzheimer's amyloid beta by binding to it

**Figure 3:**
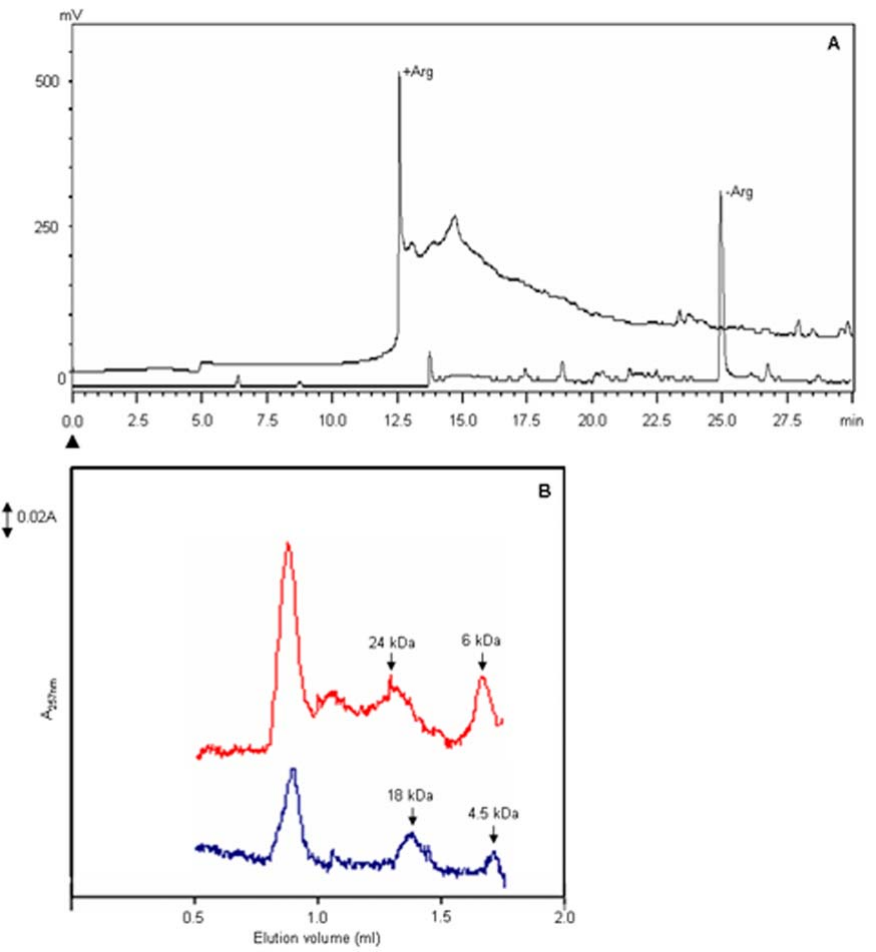
Arginine modulates the chromatographic profile of Aβ_1-42_. (A) Reverse phase chromatography of Aβ_1-42_ peptide. 10 µg of peptide was chromatographed on the RPC C8 column (250×4.6 mm) in the presence and absence arginine. The peptide was eluted with 0–60% acetonitrile linear gradient in PB at a flow rate of 0.7 ml/h and monitored at 257 nm. The arrowhead indicates the start of the gradient. The profiles in the presence and absence of arginine are indicated. (B) Size exclusion chromatography. 10 µg of Aβ_1-42_ was chromatographed on SMART Superdex G-75 column with and without arginine. The monomeric and tetrameric forms of Aβ_1-42_ elutes with larger hydrodynamic volume in the presence of arginine (red curve) compared with the control (blue curve). (The molecular weights are indicated by arrows).

The hydrophobic environment on the arginine clusters enhanced the hydrotropy of pyrene and caused an increased intensity and a blue-shift in the fluorescence emission maximum of ANS. Arginine has been reported for its hydrotropic effect with wide ranging molecules such as fatty acids [24] and many processes involving proteins, such as denaturation [15], folding [18], stability [41] and solubility [42] and peptide solubility [this study]. The involvement of hydrophobic surfaces is common to all these processes. If arginine can reduce the aggregation induced by hydrophobic surfaces, then the arginine clusters should reduce the overall hydrophobicity of the molecules. We have used Alzheimer's amyloid beta peptide (mouse Aβ_1-42_) as a model system to study the hydrophobic effect of arginine on the interactions involving hydrophobic surfaces. Aβ_1-42_ forms protofibrils by hydrophobic interactions. The protofibrils associate to give typical amyloid fibrils [43]. The reduction in the hydrophobic character of Aβ_1-42_ should be reflected in a changed profile in reverse phase chromatography (RPC), its solubility and aggregation properties. With its hydrophobic regions masked by arginine clusters, the Aβ_1-42_ should have shorter retention time in RPC in the presence of arginine. In the presence of arginine, the peptide had a shorter retention time (12.5 min, 20% acetonitrile) in the RPC C8 column (Figure 3A) as compared to the peptide without arginine (25 min, 40% acetonitrile). Elution of Aβ_1-42_ peptide in the early phase of non-polar gradient in the RPC column indicates the less hydrophobic interactions with the C8 column. Thus, arginine reduces the overall-hydrophobicity of the Aβ_1-42_ molecule.

To mask the hydrophobic surfaces of Aβ_1-42_, the arginine has to bind to these surfaces. Arginine exists in clusters and these clusters have a hydrophobic surface. It is expected that arginine clusters bind to Aβ_1-42_. Under these conditions the hydrodynamic volume of Aβ_1-42_ should increase significantly. The size exclusion chromatographic experiments showed that the monomeric form of Aβ_1-42_ eluted with a mass corresponding to 6.0 kDa in the presence of 0.2 M arginine as compared to 4.5 kDa without arginine (Figure 3B). This indicated that nine arginine molecules have bound to a single Aβ_1-42_ molecule. The 42 mer form of Aβ is known to form tetramer more predominantly than dimer or trimer [44]. We observed mostly monomeric and tetrameric forms. The tetrameric form in the presence of arginine was larger than the control by 6.0 kDa. This corresponded to an increase in molecular mass equivalent to 36 arginine molecules. The largest peak corresponds to the void volume fraction. These results clearly show that a large number of arginine molecules bind to Aβ_1-42_ to mask the hydrophobic surfaces. This experiment can not be carried out at higher temperatures, since at higher temperatures Aβ_1-42_ aggregates at faster rates [45].

### Arginine increases Aβ_1-42_ solubility and decreases fibrillar formation

**Figure 4:**
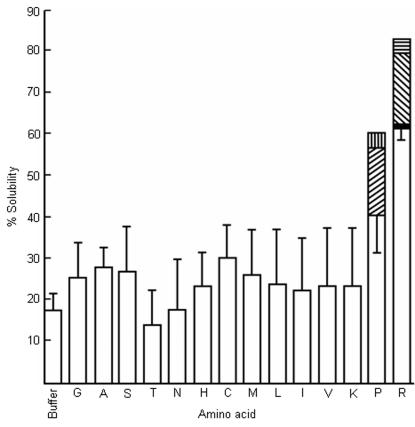
The effect of amino acids on the solubility of Aβ_1-42_. The amino acids are indicated by single letter code. Data are given as mean±SE of four experiments in duplicates with 0.2 M amino acids. Diagonal upward bar - 0.5 M proline; vertical bar - 1.0 M proline (mean of 2 experiments). With arginine, dark shade - 0.3 M arginine; diagonal downward bar - 0.4 M arginine; horizontal bar - 0.5 M arginine (mean of 2 experiments).

**Figure 5:**
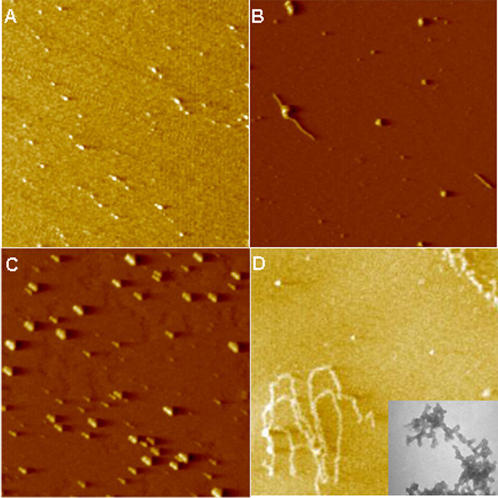
Inhibition of Aβ_1-42_ fibril formation by arginine and praline. AFM images (3×3 micron). Aβ_1-42_ was incubated in PB at 25°C for 24 h. (A) Inhibition of aggregation of Aβ_1-42_ by 0.2 M arginine. (B) Inhibition of aggregation of Aβ_1-42_ by 0.2 M proline. (C) Complete solubilization of Aβ_1-42_ at pH 10.5. No fibrils were observed. (D) Control experiment in which the fibrils formed. (Inset) Transmission electron micrograph of 24 h control sample at higher magnification (22,000×) showing spherical aggregating units.

One of the consequences of interactions between Aβ_1-42_ and clusters of arginine molecules should be the increased hydrotropy of the peptide in presence of arginine as with pyrene and decreased aggregation as with proteins. We have determined the Aβ solubility and aggregation in the presence of various amino acids. Arginine and proline were the two amino acids that enhanced the solubility of Aβ_1-42_ significantly (Figure 4) and decreased the Aβ_1-42_ aggregation in aqueous medium (Figure 5A, 5B). There was a parallelism between the solubility and inhibition of aggregation. The amino acids having no effect on the solubility did not prevent aggregation either (Figure S3). At equimolar concentrations, arginine was more effective than proline. These observations are analogous to the inhibition of aggregation of proteins due to hydrophobic forces. If it were the hydrophobic environment presented by the molecular assembly of these two amino acids that is responsible for the hydrotropic and anti-aggregation effect, then the nonpolar amino acids would be expected to be more effective. On the contrary, our results showed that nonpolar aliphatic amino acids were not effective either in increasing the solubility (Figure 4) or in inhibiting the Aβ_1-42_ aggregation, in particular (Figure S3, A, B and C) and the protein aggregation, in general [15]. The intensity of ANS fluorescence was not affected by the presence of amino acids having long aliphatic chains and nonpolar amino acids (up to 0.2 M) (Figure S4). It has been shown using model systems that ANS binds preferentially to arginine than any other basic amino acids [33]. These amino acids did not display an equal propensity to form clusters in aqueous medium as arginine and proline [[34], [35], Figure S1].

The aggregation of unfolded proteins and amyloid type proteins involve hydrophobic surfaces. In such cases, interactions of the hydrophobic surfaces provided by the clusters of arginine or proline would be more effective than the interaction between protein hydrophobic surface and an individual molecule of these two amino acids. Secondly, a hydrophobic surface of large dimensions cannot be maintained in an aqueous environment. The crystal structures of arginine and proline revealed the presence of hydrophobic columns along one of its crystallographic axis [38], [46]. Spectroscopic experiments have also demonstrated the presence of such assemblies in solutions [[34], [35]; present study]. Small amino acids do not have enough methylene groups that could provide a hydrophobic surface. The long-chain (lysine, methionine) amino acids have not been observed to have stacking interactions even at supersaturating concentrations during crystallizations [37], [47]–[49]. This could be due to the absence of side chain groups, which can have strong, multiple interactions and form a planar structure such as guanidium group. Multiple polar interactions and planar structures of the side chains help in stacking and having strong interactions with neighboring molecules in aqueous medium. The side chains of other amino acids are not aligned parallel to each other in their crystal-packing, ruling out the possibility of stacking. We also did not observe an increase in ANS fluorescence intensity in the presence of these amino acids indicating the absence of any hydrophobic surfaces. In the crystal structure of nonpolar amino acids, it is seen that their side chains are not parallel, have different conformations and side chains do not stack [50]–[52]. Without stacking, tail-to-tail interactions of these amino acids alone will be very weak to maintain a large molecular assembly to present a hydrophobic surface in aqueous medium. As these amino acids are polar at one end only, they are expected to form micellar structures rather than an open-ended bilayer structure in aqueous solution. In micellar structures, the hydrophobic moieties are not exposed to bulk aqueous medium. Hence, they are not available for interaction with the hydrophobic surfaces of the proteins. With aromatic amino acids, the dimensions of the side chains, thereby the hydrophobic surface may be too large to be exposed to water. These observations are based on the crystallographic studies of amino acids and their complexes with other ions and amino acids. These studies do not show any evidence of stacking interactions except for arginine and proline. There are crystallographic studies reporting stacking interactions between leucine residues when free leucine binds to the binding site of leucine/valine/isoleucine binding protein [53]. This may not be an analogous situation of leucine molecules interacting in aqueous solutions. The leucine and other amino acids in the binding site of the protein have fixed molecular orientations such that the interacting free ligand does not have many conformational probabilities. With the exclusion of water during binding, the interactions are quite unlike the interactions in water. We have observed that at 55°C, the fluorescence intensity (Figure 1C) and the light scattering (Figure 2D) have reached almost the minimum. In the same temperature range, proline also loses its inhibitory effect on protein aggregation [46]. In this temperature range, the polar interactions are affected more than the hydrophobic ones. Perhaps the cluster formations by these amino acids using polar interactions are affected thereby eliminating the anti-aggregation effect. It is common to find that the aggregates of small molecules and not the monomeric forms as the biologically active entities. The mechanism of action of detergents is well known in many applications. The other example is the nuclear aggregates of polyamines [54]. It is the aggregates and not the monomeric forms of polyamines that protect the genomic DNA against DNase I [55].

Our results show that arginine presents a hydrophobic environment in solutions, exists in supramolecular assemblies and binds to Aβ_1-42_. This binding modulates the hydrophobicity of Aβ_1-42_ molecule and suppresses fibrillar formation.

## Materials and Methods

### Synthesis of Aβ_1-42_

Mouse Aβ_1-42_, DAEFGHDSGFEVRHQKLVFFAEDVGSNKGAII GLMVGGVVIA was synthesized using Fmoc chemistry on an automated peptide synthesizer (model PS3, Protein Technologies, USA). The peptide was purified on a ProRPC C-18 column in a FPLC system. The peptide was stored at −20°C as lyophilized powder. Before use, the peptide was dissolved in 0.01 M NaOH and centrifuged at 16,000 g for 10 min at 4°C.

### Pyrene solubility

The solubility of pyrene was measured at various concentrations of arginine. Arginine at indicated concentrations in PB was incubated with 1 mg of pyrene at 25°C for 24 h. The solutions were centrifuged at 16,000 g for 15 min at 25°C. The absorbance of the supernatant was measured at 350 nm.

### Fluorescence spectroscopy

The changes in the emission wavelength maximum and fluorescence intensity of 1-anilino-8-naphthalene sulfonic acid (ANS) were measured in the presence of various concentrations of arginine, lysine, methionine and leucine in PB at 25°C. The fluorescence measurements were made using a Varian Cary Eclipse fluorescence spectrophotometer (Varian, USA). The excitation was at 400 nm and the emission spectra were recorded from 450–600 nm with a bandwidth of 5 nm. Blanks contained only the amino acids at the corresponding concentrations.

The temperature dependence of ANS fluorescence in presence of 0.2 M arginine were measured by changing the cuvette temperature by circulating water, maintained at different temperatures with an accuracy of ±0.1°C. The cuvettes were allowed to thermally equilibrate for 5 min before taking the reading.

### Mass spectroscopy

The amino acid solutions were prepared in MilliQ water and the pH was adjusted with dilute H_3_PO_4_ or PB. The concentration of the amino acid was 0.2 M. All mass spectra were obtained using a nanospray ESI-Q-TOF mass spectrometer (QStar XL, Applied Biosystems Inc., USA). The signal was tuned on the protonated dimer of arginine clusters. Tuning on higher-order clusters did not result in either the signal strength or change in the distribution of clusters. The settings used in this study were as follows: curtain gas flow 0.70 ml/min; the ion spray voltage 900 V; the declustering potentials DP1 100 V and DP2, 12 V; the focusing potential 100 V. The positive ion spectra were obtained for 5 min in acquire mode. Protonated dimers and trimers always appeared along with protonated monomers.

### Light scattering

Rayleigh scattering of 400 nm light was measured at 90° geometry on a Jasco J-810 spectrometer fitted with a Jasco FMO427 fluorescence emission monochromator attachment. The excitation monochromator was set at 400 nm and the emission was scanned between 385 nm and 410 nm, with the bandpass set at 10 nm for both monochromators. For room temperature measurements, five scans were performed for each sample and the measurement at 400 nm was noted and averaged. Arginine solutions were prepared in PB at indicated concentrations. Triplicate samples were used at each concentration. Similarly, for measurements at different temperatures, the peltier attachment of the spectrometer was set to the desired temperature before the performance of the five scans.

### Reverse Phase Chromatography

The Aβ_1-42_ peptide was dissolved in 0.01 M NaOH and centrifuged at 16,000 g for 10 min at room temperature. The pH was adjusted to 7.4 by the addition of PB with and without 0.2 M arginine. The final concentration of the peptide was 200 µg/ml. The solutions were incubated for 3 h at room temperature. 0.05 ml of the solution was loaded on to the RPC C8 column (250×4.6 mm) (Phenomenex, USA) equilibrated with PB using a Shimadzu HPLC set up (Model SCL-10 AVP, Shimadzu, Japan). A linear gradient of acetonitrile from 0% to 60% in 30 min was applied at a flow rate of 0.7 ml/min. For the treated sample, the equilibration and elution buffers contained 0.2 M arginine. The sample was monitored at 257 nm.

### Size exclusion chromatography

Chromatography was performed on a SMART analytical Superdex G75 column on a SMART system from Amersham Pharmacia (30 cm length, bed volume 2.4 ml) using a flow rate of 100 µl/min and with monitoring of absorption of elution carried out at 257 nm (corresponding to the absorption of phenylalanine). The column was equilibrated with PB or with PB containing 0.2 M arginine before loading of peptide samples (10 µg in 50 µl). The peptide was incubated for 1 h with 0.2 M arginine before loading.

### Aβ_1-42_ solubility measurements

The L-amino acids (Sigma Chemical Co., USA) in 10 mM phosphate buffer, pH 7.4 (PB) were added to alkali-solubilized Aβ_1-42_ to give a final concentration of 0.2 M amino acids and 10 µM Aβ_1-42_. After 30 min incubation at 25°C, the tubes were centrifuged at 16,000 g for 15 min. The supernatant was made alkaline by the addition of 0.05 M NaOH. The absorbance at 257 nm was read for the supernatant fractions (Lambda 25 model, Perkin Elmer, USA). The absorbance was compared with the 10 µM Aβ_1-42_ in 0.01 M NaOH. 0.2 M solutions of tyrosine, tryptophan, glutamic acid and phenylalanine could not be prepared due to their insolubility in PB.

### Atomic Force Microscopy (AFM)

All images were obtained in the MAC mode to ensure minimum sample damage using a PicoSPM equipment (Molecular Imaging, USA). AuCr coated MAC cantilevers, 225 µm long, resonance frequency of 83 kHz and force constant of 2.8 N/m were used for imaging. Scan speed used in was 1 line/sec. 2 µl of 10 µM Aβ_1-42_ solution with and without 0.2 M amino acids was deposited on a freshly cleaved piece of mica (1×1 cm) and allowed to stand for 2 min. Imaging was carried out in air. Minimum image processing (first order flattening and brightness contrast) was used.

### Transmission electron microscopy (TEM)

Aβ_1-42_ at 10 µM concentration in PB was incubated for 24 h at 25°C. The samples were agitated gently before being spotted on a 400-mesh carbon-coated EM grid for two minutes and stained with 1% uranyl acetate for 1 min. Micrographs were recorded using transmission electron microscope (Morgagni 268D, FEI-Philips, USA).

### Crystal packing diagram

The coordinates were taken from Karle and Karle [38] and visualized using the program Mercury (Version 1.4) [56]. The view is along the b-axis.

## Supporting Information

Figure S1 Mass spectra of methionine, lysine, leucine and proline. 0.2 M solutions in PB were used. The scan conditions were the same as used for arginine (Figure 2). (A) methionine, (B) lysine and (C) leucine do not display higher order clustering as proline (D) or arginine (Figure 2).
(9.27 MB TIF)

Figure S2 The crystal packing of arginine molecule shown in sphere model. The yellow color indicates the hydrophobic regions of arginine and the solvent molecules were shown in orange color. The coordinates were taken from Karle and Karle (see ref) and visualized using the program Mercury (Version 1.4). The view is along the b-axis.
(0.17 MB TIF)

Figure S3 AFM images of Aβ_1-42_ in the presence of 0.2 M amino acids. (A) methionine after 24 h; (B) leucine after 24 h; (C) lysine after 24 h. (D) arginine after 48 h. Only arginine prevents Aβ_1-42_ aggregation significantly. Legend as in the figure 5.
(2.45 MB TIF)

Figure S4 ANS fluorescence in presence of arginine, methionine, lysine and leucine. The excitation wavelength was 400 nm. ANS fluorescent intensity was measured at the emission λmax for the respective amino acids at different concentrations. ANS was present at 250 µM concentration. The amino acids of respective concentration formed the control. Legend as in figure 1.
(8.49 MB TIF)
