# Supplementary figures and images for: Inhibition of Protein Aggregation: Supramolecular Assemblies of Arginine Hold the Key
Source: PLoS One. 2007 Nov 14;2(11):e1176. doi: 10.1371/journal.pone.0001176 (PMC2064962; doi:10.1371/journal.pone.0001176)

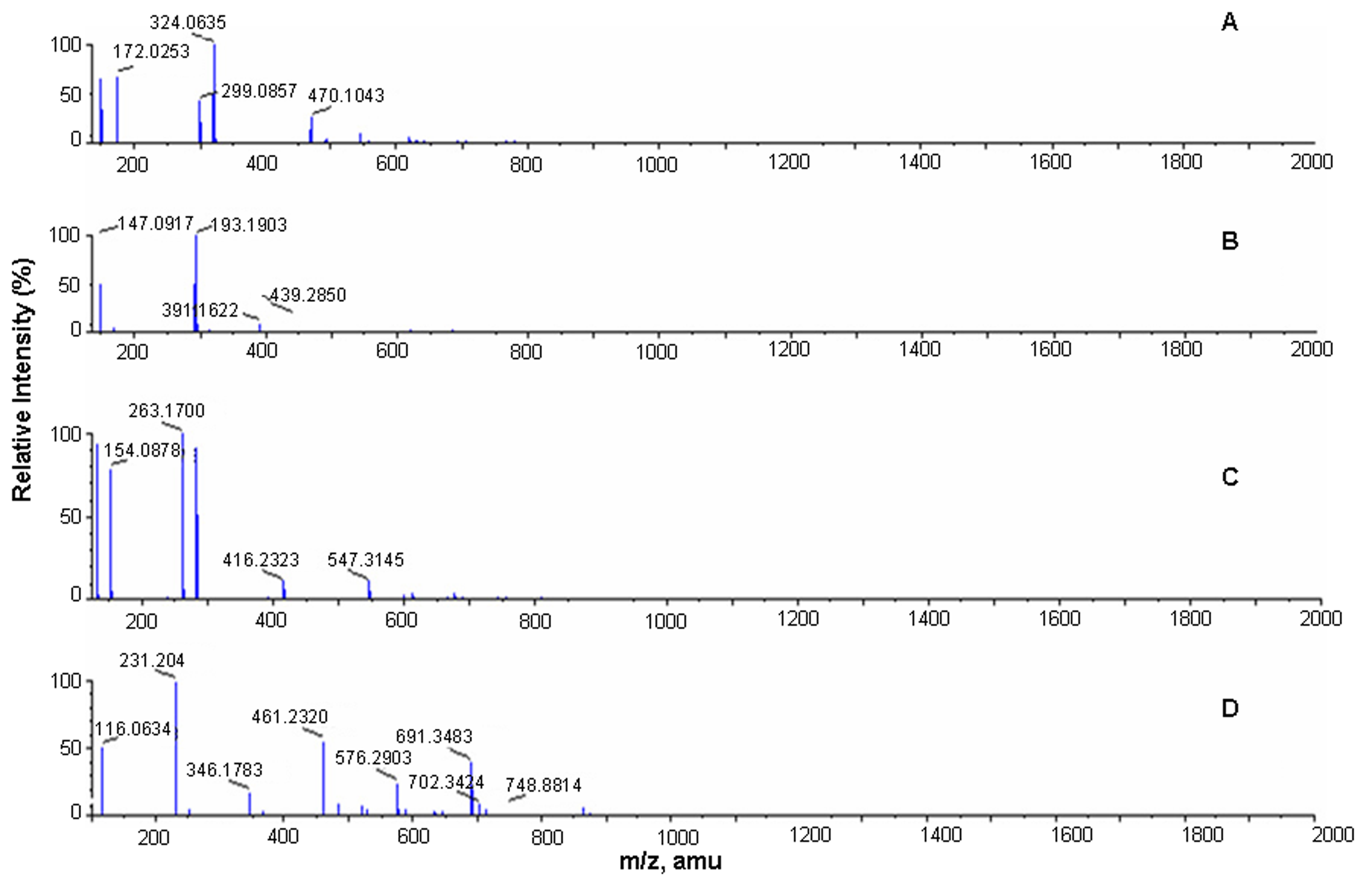

Supplement: Figure S1 — Mass spectra of methionine, lysine, leucine and proline. 0.2 M solutions in PB were used. The scan conditions were the same as used for arginine (Figure 2). (A) methionine, (B) lysine and (C) leucine do not display higher order clustering as proline (D) or arginine (Figure 2). (9.27 MB TIF) [file pone.0001176.s001.tif]

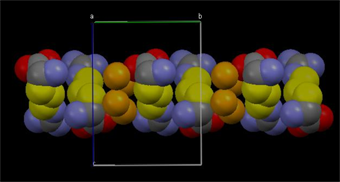

Supplement: Figure S2 — The crystal packing of arginine molecule shown in sphere model. The yellow color indicates the hydrophobic regions of arginine and the solvent molecules were shown in orange color. The coordinates were taken from Karle and Karle (see ref) and visualized using the program Mercury (Version 1.4). The view is along the b-axis. (0.17 MB TIF) [file pone.0001176.s002.tif]

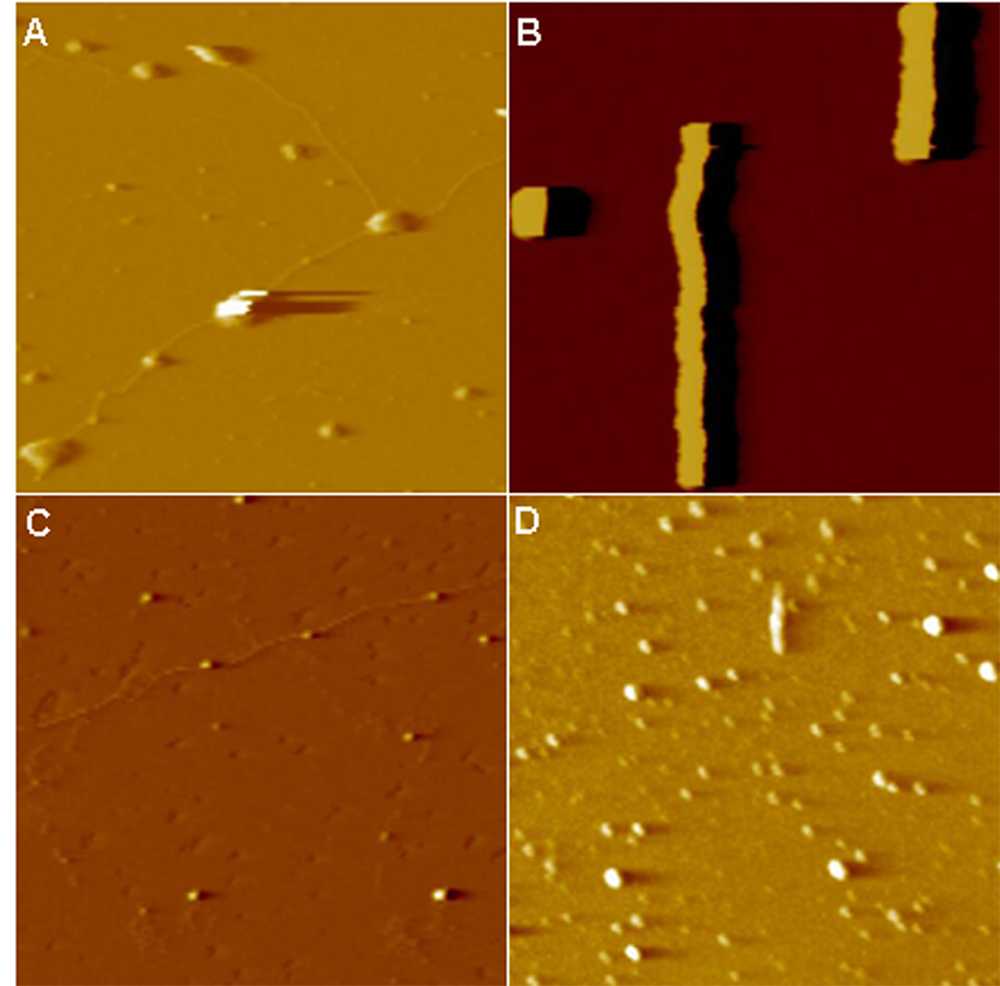

Supplement: Figure S3 — AFM images of Aβ1-42 in the presence of 0.2 M amino acids. (A) methionine after 24 h; (B) leucine after 24 h; (C) lysine after 24 h. (D) arginine after 48 h. Only arginine prevents Aβ1-42 aggregation significantly. Legend as in the figure 5. (2.45 MB TIF) [file pone.0001176.s003.tif]

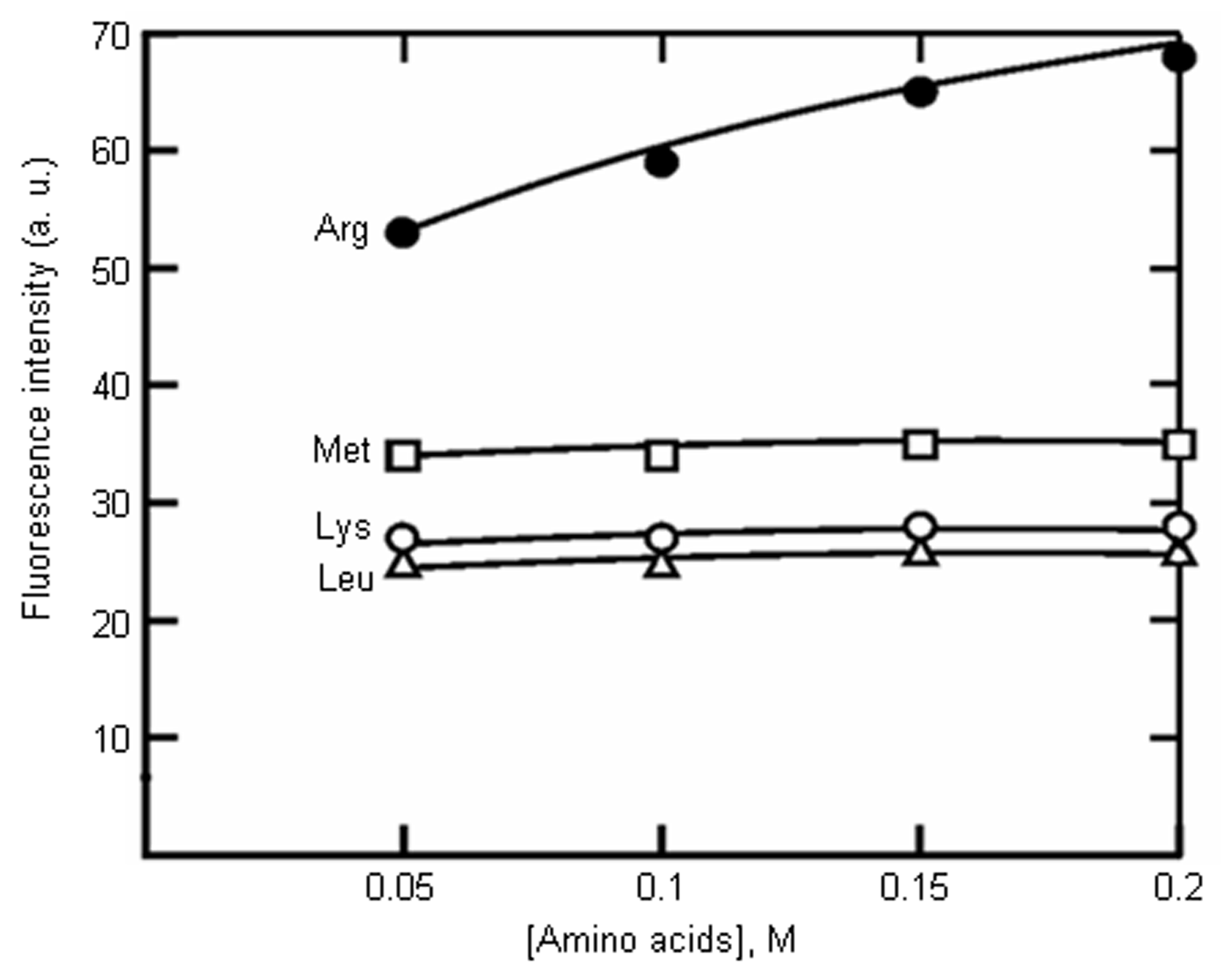

Supplement: Figure S4 — ANS fluorescence in presence of arginine, methionine, lysine and leucine. The excitation wavelength was 400 nm. ANS fluorescent intensity was measured at the emission λmax for the respective amino acids at different concentrations. ANS was present at 250 µM concentration. The amino acids of respective concentration formed the control. Legend as in figure 1. (8.49 MB TIF) [file pone.0001176.s004.tif]
